# Dynamic Behavior of Aluminum Alloy Aw 5005 Undergoing Interfacial Friction and Specimen Configuration in Split Hopkinson Pressure Bar System at High Strain Rates and Temperatures

**DOI:** 10.3390/ma13204614

**Published:** 2020-10-16

**Authors:** Amine Bendarma, Tomasz Jankowiak, Alexis Rusinek, Tomasz Lodygowski, Bin Jia, María Henar Miguélez, Maciej Klosak

**Affiliations:** 1 Laboratoire d’Innovation Durable et de Recherche Appliquée (L.I.D.R.A), Universiapolis, Bab Al Madina, Qr Tilila, 80000 Agadir, Morocco; maciej.klosak@gmail.com; 2 Institute of Structural Analysis, Poznan University of Technology, Piotrowo 5, 60-965 Poznań, Poland; tomasz.jankowiak@put.poznan.pl; 3 Laboratory of Microstructure Studies and Mechanics of Materials, UMR-CNRS 7239, Lorraine University, 7 rue Félix Savart, BP 15082, 57073 Metz CEDEX 03, France; alexis.rusinek@univ-lorraine.fr; 4 Chair of Excellence Universidad Carlos III de Madrid, Department of Mechanical Engineering, Avda. de la Universidad 30, 28911 Leganés, Madrid, Spain; mhmiguel@ing.uc3m.es; 5 Institute of Combustion Engines and Powertrains, Poznan University of Technology, Piotrowo 3 Street, 60-965 Poznan, Poland; tomasz.lodygowski@put.poznan.pl; 6 ENSAM-Arts et Métiers ParisTech 4 Rue Augustin Fresnel, 57070 Metz, France; jiabin@mail.sdu.edu.cn

**Keywords:** split Hopkinson pressure bar, aluminum alloy, dynamic friction, experiment, specimen configuration, numerical simulation

## Abstract

In this paper, experimental and numerical results of an aluminum alloy’s mechanical behavior are discussed. Over a wide range of strain rates (10^−4^ s^−1^ ≤ έ ≤ 10^3^ s^−1^) the influence of the loading impact, velocity and temperature on the dynamic response of the material was analyzed. The interface friction effect on the material’s dynamic response is examined using a split Hopkinson pressure bar (SHPB) in a high temperature experiment using finite element analysis (FEA). The effect of different friction conditions between the specimen and the transmitted/incident bars in the SHPB system was examined using cylinder bulk specimens and cylinder plates defined with four-layer configurations. The results of these tests alongside the presented numerical simulations allow a better understanding of the phenomenon and reduces (minimizes) errors during compression tests at high and low strain rates with temperatures ranging from 21 to 300 °C.

## 1. Introduction

The experimental techniques used for the mechanical characterization of materials are widely diverse. The choice of it is not accidental; they target specific objectives, according to the needs of industry whether in the civil or military field. These techniques can be divided into two main categories:

Techniques using hydraulic machines allowing stress at low and medium strain rates or techniques based on the bars device of the split Hopkinson pressure bar (or Kolsky bars [1]) to achieve high strain rates up to 10^−4^ × s^−1^.

As for the second category, these techniques are much varied and can be classified into three most commonly used versions, namely: compression, tension and torsion.

To define the behavior of materials to be used in industrial applications, it is necessary to perform several tests to understand the different loading effects of strain rates and temperatures on the material’s behavior. As shown in Table 1, several techniques have been used to cover a wide range of strain rates.

Interfacial friction is an important element in the split Hopkinson pressure bar (SHPB) testing process for determining the stress condition of the specimen. This has a negative influence on the most important uniaxial stress state theory, and thus lubricant is commonly used at the interfaces to prevent multiaxial stress states [2,3]. Through theories and experiments it is revealed that the material Poisson’s ratio, the friction coefficient, the specimen’s length-to-diameter ratio and the axial strains are the major factors influencing interfacial friction results [4,5,6,7,8,9,10,11]. For instance, in SHPB research, Brisco and Nosker [12] studied the effect of the interface friction on the yield behavior of a high density polyethylene when this one is compressively marred at high strain rates equivalent to 25% at high strain rates (10^3^ × s^−1^) in SHPB experiments. Lu and Zhang [13] concluded that the SHPB test analysis and correction protocol should be done on the basis of a kinetic friction model. The dynamic friction behavior of polycarbonate at room temperature and a low one of −60 °C was examined by Troutman et al. [14] using a split Hopkinson pressure bar and utilizing specimens with different thicknesses. There was also mention of the various effects of two forms of lubricating oils, polytetrafluoroethylene and molybdenum disulfide. In the literature, there have been many studies focused on the measurement and adjustment of the unconventional SHPB test. Lu and Zhang [13] studied the discrepancy between constant and kinetic friction models, which could be the cause of different results, and it was proposed that the procedure could be performed by correcting the SHPB test based on a kinetic friction model.

Long Zhang et al. [15] studied the correlation between dynamic compression behavior and the microstructure of 6005 aluminum alloy artificially aged at elevated temperatures at a wide range of temperatures from 180 to 330 °C. Additionally, they observed the increase of energy absorption while there was no evident changes in the dynamic stress strain behavior with increasing strain rate. J.J. Gracio et al. [16] investigated the artificial aging and shear deformation behavior of the 6022-T4 alloy at a wide range of temperatures from 160 to 260 °C, using TEM and XRD analyses, it has been showed that the 6022-T4 alloy can be substantially hardened through a short aging treatment at temperatures in excess of 200 °C and that the T4 and under-aged conditions lead to permanent softening of the flow stress. Y. Chen et al. [17] studied the stress–strain behavior of extruded AA6xxx and AA7xxx aluminum alloys in T6 temper at wide strain rate ranges, using a split Hopkinson tension bar, they found that the AA6xxx has no significant rate sensitivity in the stress–strain behavior. On the other hand reasonable rate sensitivity was found for the AA7xxx alloys. R. Smerd et al. [18] identified the constitutive response and damage evolution in the automotive aluminum alloy sheet the AA5754 and AA5182 aluminum alloy at high strain rates, room temperature and elevated temperature experiments, they used a tensile split Hopkinson bar, to minimize the geometry effects a modified specimen has been used, the Johnson–Cook constitutive parameters model has been identified and the model validate well the flow stress for both aluminum alloys AA5754 and AA5182 and the strain rate parameter was found to be low in comparison to other alloys. Ketill O et al. [19] investigated the fracture behavior of the aluminum alloy AA7075-T651 at quasi-static and dynamic loading using different stress states. Using a blunt and conical projectile they found that the stress within the perforation tests is way too complex, and the strain rate significantly higher than the tensile and compression tests, and they found also that when the material is tested in quasi-static loading in the uniaxial tensile test, the fracture strain depends on the orientation of the tensile specimen in the rolling of the plate. Whereas the 45° orientation incorporates an essentially larger ductility.

## 2. Dynamic Mechanical Properties and Constitutive Model of AW 5005

### 2.1. Aluminum AW 5005 Description

In this work, a study on aluminum alloy AW5005 was reported. Within marine atmospheres, this alloy included principally 0.8 percent magnesium and had medium strength, good weldability and reasonable corrosion resistance. The metallurgical state of the aluminum alloy used in this work was as received, Table 2 and Table 3. Compared to other aluminum alloys, it had a lower density and a better thermal conductivity. It is the aluminum type most widely found in sheet and plate types [20,21,22]. The EN AW 5005 aluminum alloy was investigated based on our experimental results.

### 2.2. Material Parameters

Using the Johnson–Cook’s experimental tests parameters, the Johnson Cook (JC) model [23] was identified. The thermo-viscoplastic behavior of the AW5005 aluminum alloy is defined as described in Equation (1).

(1) $$\sigma =\left(A+B\varepsilon_{pl}^{n}\right)\left(1+Cln\frac{{\dot{\bar{\varepsilon }}}^{p}}{{\dot{\varepsilon }}_{0}}\right)\left(1-T^{*}{}^{m}\right)$$

where *A*, *B*, *n* and *C* are the yield stress, the strain hardening coefficients and the strain rate sensitivity coefficient respectively, ${\dot{\varepsilon }}_{0}$ is the reference strain rate and m is the temperature sensitivity parameter. In this work, adiabatic conditions were assumed. Therefore, the last term of the JC model related to the non-dimensional temperature where $T^{*}$ is the homologous temperature.

(2) $$T^{*}=\left(\frac{T-T_{0}}{T_{m}-T_{0}}\right)$$

where $T_{m}$ and $T_{0}$ are the melting and room temperatures respectively.

The material parameters were obtained from experiments. Parameter C was calculated using results under quasi-static loading (strain rates from 0.001 to 0.15 s^−1^). In this range, small strain rates sensitivity was observed. Using the minimum least square optimization method value of C, which was 0.003. These constants are shown in Table 4 [21].

**Figure 1:**
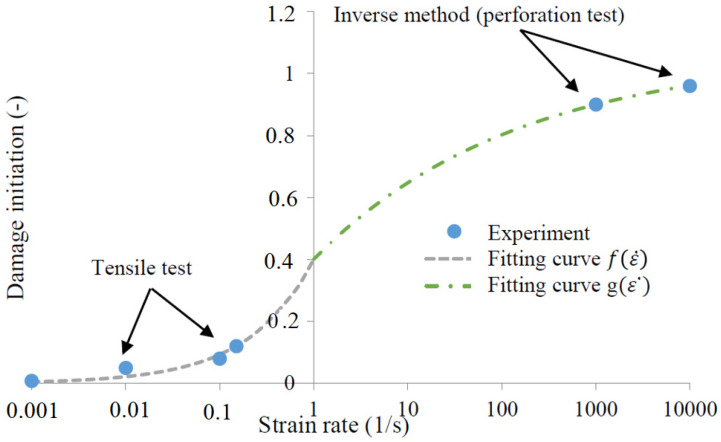
Plot of the failure strain versus plastic strain rate using the optimized model [21].

In order to completely define the material behavior, a failure criterion was proposed by Bendarma et al. [21], and it helps to reflect a failure mode of structures of materials. The damage initiation criterion (Equation (3)) is defined with two glued functions with four constants H, I, J and K. Using an optimization method, a good correlation over a wide range of strain rates was obtained, see Figure 1.

(3) $$\varepsilon_{\;D-init}^{\;pl}=\{\begin{array}{ll} f\left(\dot{\varepsilon }\right)=He^{\left(I\;log_{10}\left(\dot{\varepsilon }\right)\right)} & if\;\dot{\varepsilon }\leq {\dot{\varepsilon }}_{Transmition}\\ g\left(\dot{\varepsilon }\right)=J-\left(K\;e^{\;log_{10}\left(\dot{\varepsilon }\right)}\right) & if\;\dot{\varepsilon }\geq {\dot{\varepsilon }}_{Transmition} \end{array}$$

The estimated constants are reported in Table 5 with ${\dot{\varepsilon }}_{Transmition}$ = 1 $s^{-1}$.

## 3. Dynamic Compression Testing Using SHPB

At high strain rates, the most widely used technique for studying the dynamic properties of materials is based on the split Hopkinson pressure bar (SHPB). This method was originally developed by Kolsky [1] then it went through several improvements. Now known as split Hopkinson bars. Later modifications allowed for tensile, compressive and torsional testing. Its principle is based on the theory of the propagation of elastic waves in bars, and makes it possible to reach strain rates between 10^2^ × s^−1^ and 10^4^ × s^−1^. The principle of the Hopkinson bars is known and is exceptionally regularly utilized for testing the dynamic behavior of materials and has been the object of numerous publications, [24,25,26,27,28]. R.M. Davies et al. [29] described an electrical approach for measuring the relation between pressure and time in tests on high pressures of a brief duration using the Hopkinson’s method. The pressure is applied ordinarily to one end of a cylindrical steel bar, creating a stress pulse that gives rise to radial and longitudinal displacements within the bar. The theory was used to analyze the errors within the experiments caused by the pressure bar itself. A.G. Bazle et al. [30] presented a critical review of three classic papers by B. Hopkinson, RM Davies and H Kolsky. According to the method developed by Hopkinson, experimental techniques, experimental protocol including bar calibration, specimen configuration, pulse shaping and data analysis following the essential dispersion correction methodology are discussed. The elastic–plastic metals and methodologies for soft and hard materials are also presented. M. Quik et al. [31] analyzed the dynamic mechanical properties of automotive thin sheet steel using Hopkinson bars, different tests were carried out (tension, compression and shear) and the specimens were prepared from thin sheet steel and tested at different strain rates ranging from 10^−3^ to 10^3^ × s^−1^.

**Figure 2:**
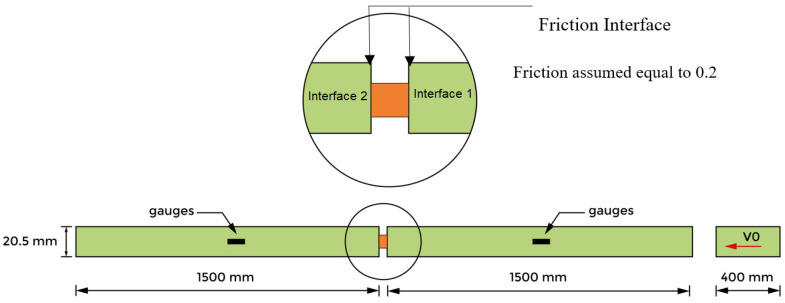
Schematic description of the Hopkinson bar device.

The used assembly for our study is illustrated in Figure 2. The specimens were placed between two identical bars of high elastic limit with respect to that of the tested material. The first and second bars are named “input bar” and “output bar” respectively. The entry bar is that of which the free end receives the longitudinal impact of a cylindrical projectile.

**Figure 3:**
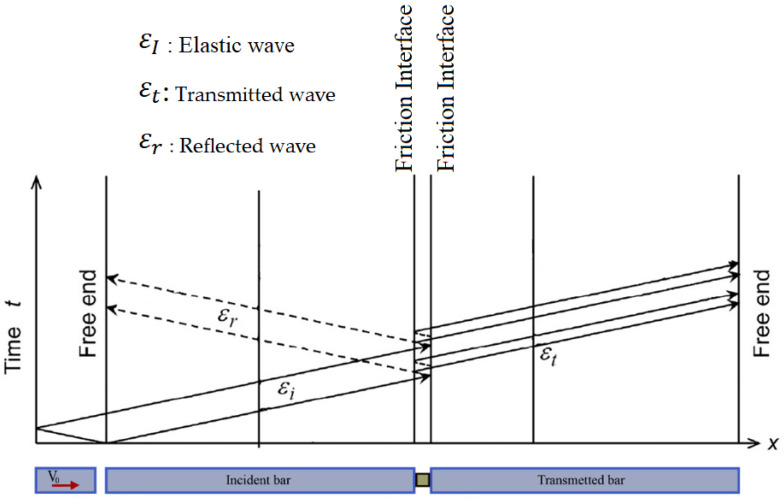
Wave propagation principle, Lagrange diagram.

An incident elastic wave, generated by the initial impact velocity of the projectile, propagates from the input bar to the specimen according to the flow diagram illustrated in Figure 3. At the contact surface of the specimen and the entry bar (interface 1), part of this wave propagates in the specimen and the other part is reflected.

The transmitted wave passes through the specimen and encounters the specimen/output bar interface (interface 2). Part of this wave is reflected and again passes through the specimen in the opposite direction. This wave propagation makes it possible to generate a dynamic deformation within the specimen. The bars are made of high strength steel having a high yield stress level (1 GPa) and a high level of hardness. It should be noted that the elastic limit and the hardness of the material constituting the bars are much greater than those of the material to be tested.

According to the Lagrange diagram, the time *t* = 0 corresponds to the moment of impact of the projectile. Strain gauges are glued on both bars to measure the incident wave produced by the impact of the projectile on the incident bar, the reflected wave at the incident bar/specimen interface and the transmitted wave to the outgoing bar at the transmitted bar/specimen interface.

The projectile launched with an initial velocity $V_{0}$ impacts the incoming bar and generates an incident elastic wave $\varepsilon_{I}$, which propagates in the bar with a celerity $C_{0}$ close to 5000 m/s for the steel bar:

(4) $$C_{0}=\;\sqrt{\frac{E}{\rho }}$$

E and ρ denote respectively the Young’s modulus and the density of the SHPB bars. Thus, the intensity of the incident stress is defined by:

(5) $$\sigma_{I}=\;\frac{1}{2}\;\rho C_{0}V_{0}\;=\;E\;\varepsilon_{I}$$

where $V_{0}$ is the initial impact velocity and $\varepsilon_{I}$ is the incident wave.

The characteristic equations associated with one-dimensional elastic wave propagation in the bar make it possible to express the particle velocities at the two interfaces:

(6) $$\{\begin{array}{c} v_{input}\left(t\right)=\;C_{0}\left(\varepsilon_{I}\left(t\right)-\varepsilon_{R}\left(t\right)\right)\\ v_{output}\left(t\right)=\;C_{0}\varepsilon_{T}\left(t\right) \end{array}$$

where $\varepsilon_{T}$ denotes the transmitted wave and $\varepsilon_{R}$ the reflected wave, $v_{input}$ and $v_{onput}$ denote respectively the input and output velocities.

The average rate of the axial strain in the specimen is given by:

(7) $$\dot{\varepsilon_{n}}=\frac{v_{input}\left(t\right)-\;v_{output}\left(t\right)}{l_{0}}=\;\frac{C_{0}}{l_{0}}\;\left(\varepsilon_{I}-\;\varepsilon_{R}-\varepsilon_{T}\right)$$

where $l_{0}$ denotes the initial length of the specimen. Since the displacement and force are known it is possible to calculate stress and strain at the two specimen/bar interfaces:

(8) $$\{\begin{array}{c} F_{input}\left(t\right)=\;A_{0}E\left[\varepsilon_{I}\left(t\right)+\;\varepsilon_{R}\left(t\right)\right]\\ F_{output}\left(t\right)=\;A_{0}E\varepsilon_{T}\left(t\right) \end{array}$$

The nominal average axial stress in the specimen is expressed as follows:

(9) $$\{\begin{array}{c} \sigma \left(t\right)=\;\frac{F_{input}\left(t\right)+F_{output}\left(t\right)}{2A_{s}}\\ \sigma \left(t\right)=\;\frac{A_{0}E\left[\varepsilon_{I}\left(t\right)+\;\varepsilon_{R}\left(t\right)+\varepsilon_{T}\left(t\right)\right]}{2A_{s}} \end{array}$$

where $A_{0}$ is the cross-section of the bar, $A_{s}$ is the cross-section of the specimen and $F_{input}$ and $F_{ouput}$ are respectively the input and output forces. The stress is supposed to be balanced if the boundary conditions are frictionless, the stress in the specimen is also uniaxial.

In other words, assuming that the stress and strain are homogeneous and the stress is uniaxial in the specimen, and the elastic wave propagation is one-dimensional (1D) without dispersion in the bars.

Once the force equilibrium is achieved ($F_{input}$ = $F_{ouput}$), the Equation (8), reduces to:

(10) $$\varepsilon_{I}\left(t\right)+\;\varepsilon_{R}\left(t\right)=\varepsilon_{T}\left(t\right)$$

In other words, assuming that:

- The stress and the strain are homogeneous in the specimen.
- The stress is uniaxial in the specimen.
- The elastic wave propagation is one-dimensional (1D) without dispersion in the bars.

Sometimes the friction on the bar/specimen interfaces may cause a change in the state of uniaxial stress and may lead to erroneous results [32]. Many authors [1,24,33,34], who worked on it have shown that the geometry of the specimen, meaning the ratio $L_{0}$/$d_{0}$ (where $d_{0}$ denotes the initial specimen diameter), determines the importance of the friction effect on the results of the test. To reduce the effect of this factor, it is recommended to lubricate the bar/specimen interfaces and set the ratio $L_{0}$/$d_{0}$ between 0.5 and 1 [32,35]. However, the friction correction method proposed by Klepaczko-Malinowski [34], which is described in the quasi-static compression section (the model is valid both statically and dynamically), should be applied.

A thorough knowledge of the behavior of metallic materials requires a more detailed analysis of the rupture mechanisms, damage and absorption of energy. The dynamic perforation has been considered a genuine choice to solve this problem.

## 4. Description of the Specimen and Hopkinson Bar Device Using the Thermal Chamber

**Figure 4:**
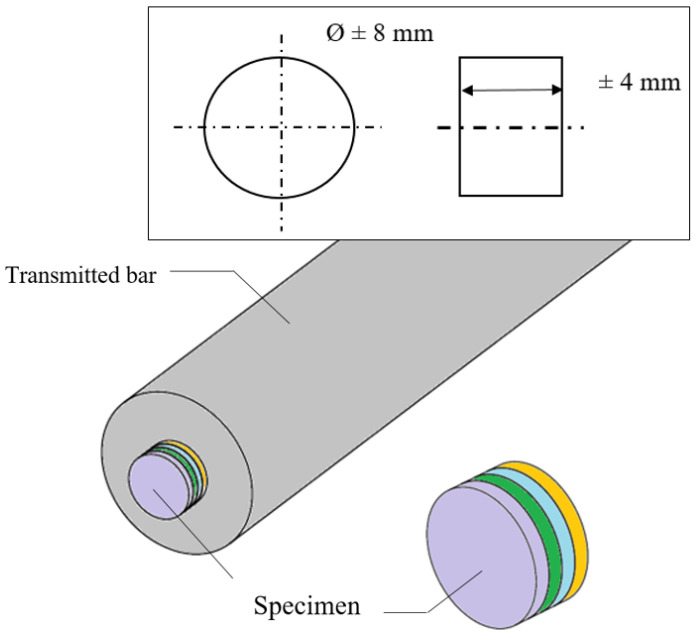
Geometry of the compression test specimen in dynamic compression.

**Figure 5:**
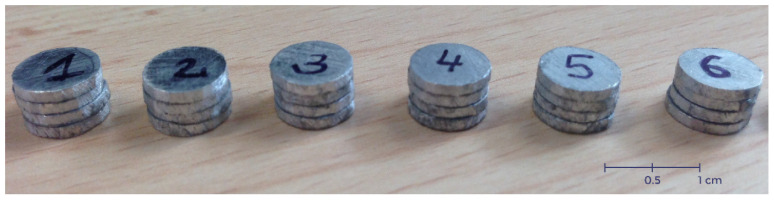
Split Hokinson pressure bar (SHPB) specimen’s built using four disks cut from the aluminum sheet of 1 mm thickness.

Cylinder sheet specimens made of aluminum alloy AW 5005 were manufactured in this configuration and put to use in the present work in order to examine the complex behavior using the SHPB system. One specimen was built using four cylinders sheet specimens of 1 mm with the following dimensions (Figure 4): a length of $L_{0}$ = 4 mm and a diameter of $\emptyset_{0}$ = 8 mm corresponding to a ratio of $S_{0}$ = 0.5 (Figure 5).

The diameter of the bar was 20.5 mm, the length of the projectile was 400 mm and the length of the bars was 1.5 m. To avoid the transmitted bar motion, rubber oil was used as cushion content. As per numerical analysis, the cylinder layer specimen consists of four identical thin cylinders with a diameter of 8 mm and a thickness of 1 mm each. To form the cylinder sheet specimen, the four thin cylinders were bound together using an adhesive.

In the latest SHPB tests for cylinder layer specimens, four initial impact velocities of 5.17 m/s, 8.78 m/s, 10.75 m/s and 13.03 m/s were recorded. Additionally, based on the reported strain details on the incident and transmitted bars, the friction coefficient considered between the bars was equal to μ = 0.2.

**Figure 6:**
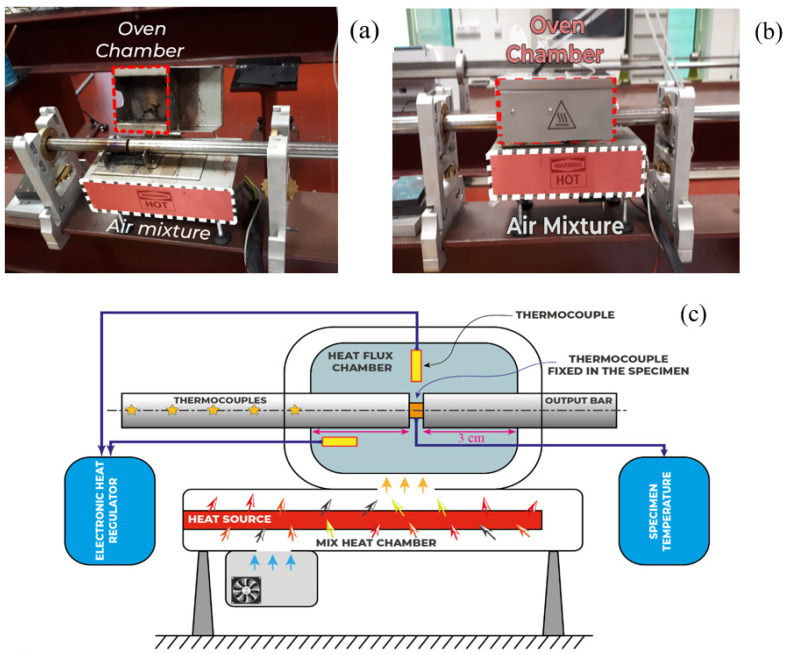
(**a**) Hopkinson pressure bars used during dynamic tests with a thermal chamber; (**b**) description of the internal part and (**c**) description of the air flowing.

In order to determine the material’s behavior at elevated strain rates (up $\dot{\varepsilon }$ ≥ 1000 $s^{-1})$ and temperature range (20 °C ≤ $T_{0}$ ≤ 170 °C), experimental investigations were performed using a particular furnace attached to the split Hopkinson pressure bars (SHPB). The device makes it possible to have a uniform temperature all over the specimen’s volume, while avoiding having a temperature gradient along the specimen as addressed by Lennon and Ramesh [36,37]. The developed and used set-up for dynamic compression tests is presented in Figure 6. Tests were carried out at a variety of strain rates, and different temperatures from room ambient temperature to 300 °C.

The specimen was 4 mm thick and had a diameter of 8 mm. A bespoke oven was designed in order to get and maintain an evenly distributed temperature, Figure 6. A constant volume was then heated to an initial temperature $T_{0}$. This volume was greater than the chamber volume where the specimen was wedged within the two bars of Hopkinson, Figure 6c [38,39]. To maintain a constant temperature in the specimen chamber, a fan was used to effuse out the needed hot air stream.

A SHPB must be utilized to define the strain rate sensitivity for strain rates greater than 5000${\;s}^{-1}$ [40,41] (Figure 2).

To study this effect based on dynamic compression tests, some points must be remembered, The first one is related to the geometric parameter $S_{0}=\;L_{0}/\emptyset_{0}$, where $L_{0}$ is the initial length and $\emptyset_{0}$ is the initial diameter of the specimen. Klepaczko et al. [34] discussed the effect of various specimen geometries under quasi-static loading, they observed that behavior varies in terms of the degree of stress while retaining the same hardening, and creates additive energy due to the influence of over stress that depends on the ratio of $S_{0}=\;L_{0}/\emptyset_{0}$. Thus even if a lubricant was used during the tests, the material behavior concluded from experiments $\sigma_{measured}$ involves a geometry related friction effect that can be defined as stated in Equation (11), in consequence, the effect of friction $\Delta \sigma_{friction}$ may be corrected given previous work [34], assuming that $\dot{\varepsilon }=Cste$, as defined in Equation (12).

The specimen surfaces in contact with the SHPB bars were lubricated with MoS2 molybdenum grease to minimize friction and avoid the barrel effect. However, even if a lubricant experiment includes the friction effect associated with the geometry that was used during the tests, the estimated behavior of the material can be obtained from the equation below:

(11) $$\sigma_{measured}=\;\sigma_{material}+\;\Delta \sigma_{friction}$$

where $\sigma_{Material}$ is the intrinsic behavior of the material without the friction effect and $\Delta \sigma_{Friction}$ is the increase in stress due to friction (coefficient of friction μ).

The same specimen geometry was used for dynamic compression tests (SHPB). The dimensions of the specimen were fixed taking into account the restrictive conditions related to the dynamic test [20]. Based on the model proposed by Klepaczko-Malinowski [34,42], the effect of friction can be corrected as follows:

(12) $$\{\begin{array}{c} \sigma_{material}=\;\sigma_{measured}-\;\Delta \sigma_{friction}\\ \sigma_{material}=\;\sigma_{measured}\left(1-\frac{\mu }{3}\frac{\emptyset_{0}}{L_{0}}\right)=\;\sigma_{measured}\left(1-\frac{\mu }{3s_{0}}\right) \end{array}$$

where μ is the coefficient of friction, $d_{0}$ and $l_{0}$ are respectively the diameter and the initial length of the specimen. This formulation is valid in the linear growth domain of Δσ with the coefficient of friction. For our specific case where $l_{0}$/$d_{0}$ = 0.5, the correction is applicable for μ ≤ 0.2 [34].

## 5. Numerical Simulations Analysis Description and Cases Considered

### 5.1. Specimen Geometry Comparison

For dynamic compression tests, a numerical simulation using the Abaqus explicit code was carried out, a comparison between two geometries (one cylinder and four cylinder sheet) was used at the start, a smaller cylinder specimen was used with the following dimensions a length of $L_{0}$ = 4 mm and a diameter of $\emptyset_{0}$ = 8 mm corresponding to a ratio of $S_{0}$ = 0.5. A smaller specimen built using four cylindrical sheet specimens of 1 mm thickness with the following dimensions: a total length of $L_{0}$ = 4 mm and a diameter of $\emptyset_{0}$ = 8 mm corresponding to a ratio of $S_{0}$ = 0.5. The four thin cylinders were glued together, a friction coefficient between the cylinders equal to 0.3 was taken into consideration during numerical simulations. To model the behavior of AW 5005 aluminum alloy, the JC model [23] using previous parameters was used taking into account hardening, strain rate and temperature sensitivity. The model of this numerical simulation contained four main parts: the projectile, the specimen and the two SHPB bars.

Numerous mesh densities were checked to achieve correct numerical results with minimum computing time and an optimum mesh with an aspect size of 0.5 mm × 0.5 mm was finally used. There were over 160,000 nodes and 130,000 hexahedral elements (type of C3D8R Abaqus) in the finite element analysis (FEA) model. For all simulations, the same measurements of the SHPB system were used, with a projectile lengths of 400 mm, incident and transmitted bar lengths of 1500 mm and bars and projectile diameters of 20.5 mm. The initial velocity of the projectile was set to be 13 m/s.

**Figure 7:**
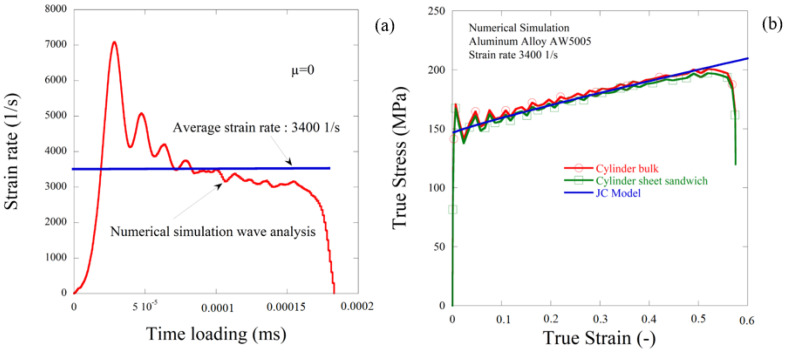
(**a**) Strain rate for an impact velocity 13 m/s and (**b**) comparison between numerical results with the JC model (without friction correction) using cylinder and cylinder sheet specimen configuration.

Results presented in Figure 7b shows a good correlation in terms of true stress and true strain using the same material parameters and conditions. Similar dynamic reaction problems were observed in both cylinder and cylindrical sheet experiments. In the other side, it is possible to take specimens with fused cylindrical layers as a uniform design to increase the controllability of the experiment for materials consisting of thin plates.

**Figure 8:**
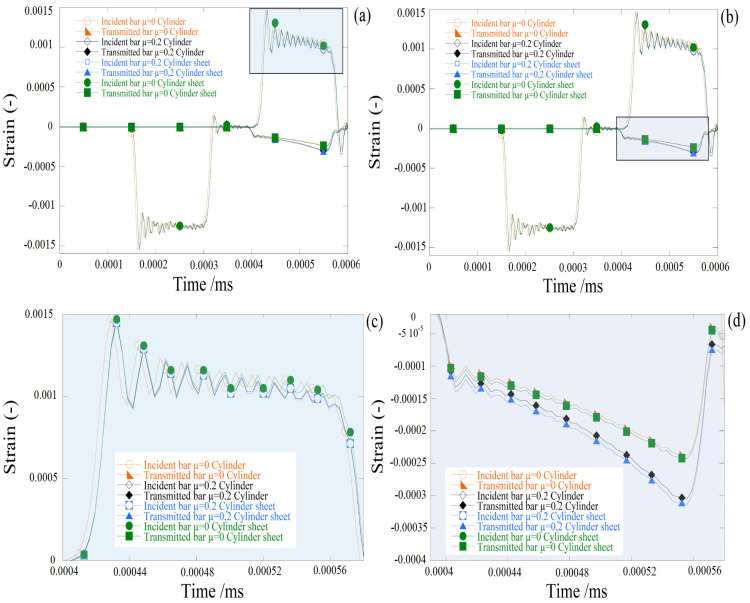
Comparison of cylinder versus cylinder sheet configuration at different friction conditions (μ = 0 and μ = 0.2). (**a**,**b**) reflected and transmitted waves (**c**) reflected strain waves and (**d**) transmitted strain waves.

In the case of the friction surface contact, a small disparity between the cylindrical bulk specimen and the cylindrical layer specimens for four different friction coefficients is seen in the contrast of the propagation of stress waves (see Figure 8, for case μ = 0.2). In other words, common axial impact reactions to equal friction interface conditions were encountered by cylindrical bulk specimens and cylindrical layer specimens.

Consequently, specimens with cylindrical layers with reasonable friction between the layers can be considered as a reliable composition in the Hopkinson compression experiment when it is difficult to cut the raw materials into a desirable length (depth) cylindrical specimen. To achieve optimum specimen depth, thin cylinder plates can be cut and assembled, resulting in the correct desired result similar to a single layer cylinder.

### 5.2. Friction Effect under Dynamic Compression

**Figure 9:**
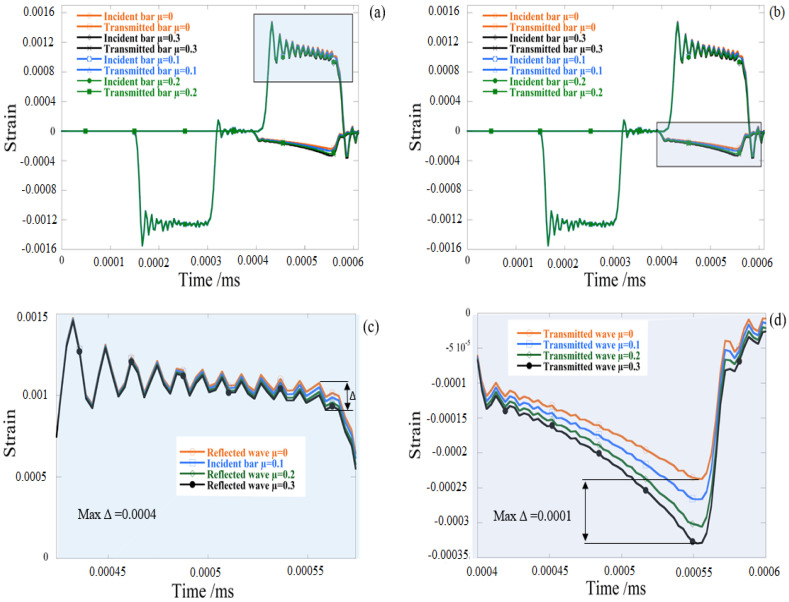
Comparison of reflected and transmitted waves under different friction conditions (cylinder sheet). (**a**,**b**) reflected and transmitted waves (**c**) reflected strain waves and (**d**) transmitted strain waves.

**Figure 10:**
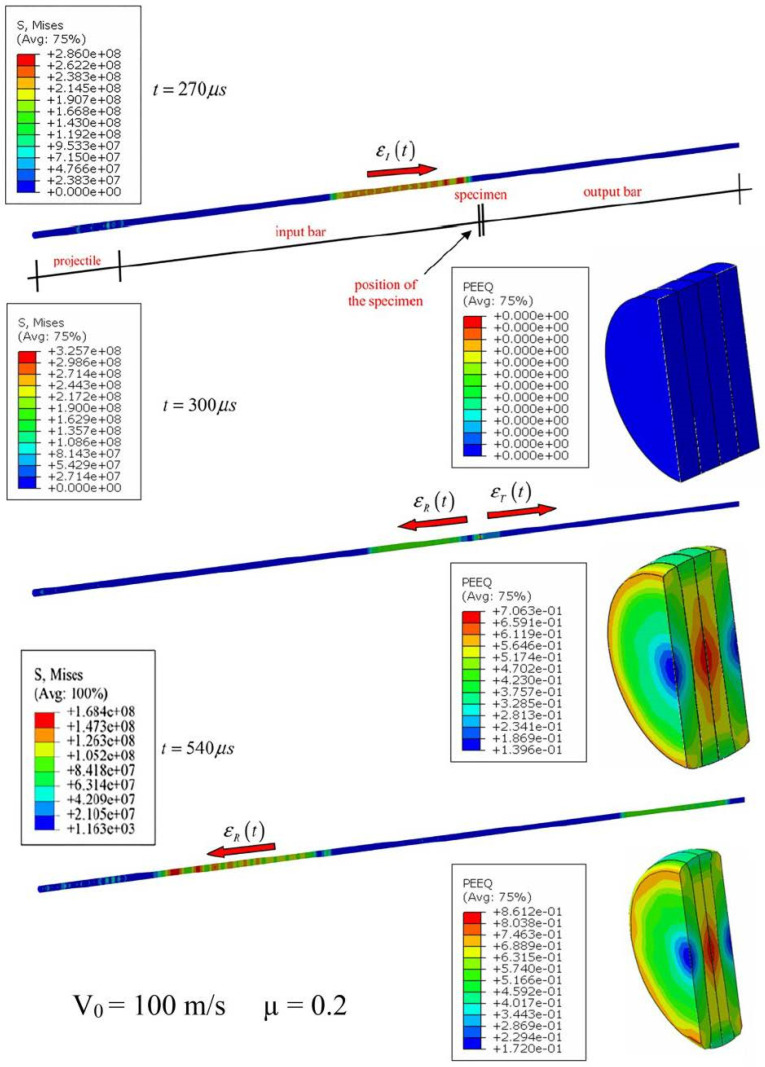
Numerical simulation of the wave’s propagation along SHPB with the AW 5005 aluminum alloy as received specimen.

The influence of friction and temperature on incident/transmitted bar pulses was studied with varying friction conditions using the same experiment specimen configurations. During the FEA numerical simulations, axial strain curves along the incident and transmitted bars at the location of the gauge were reported as shown in the Figure 9. Based on numerical measures, a thorough study of friction and temperature effects was given. Furthermore, in Figure 10, the process of elastic wave propagation could be observed during the dynamic impact in SHPB.

**Figure 11:**
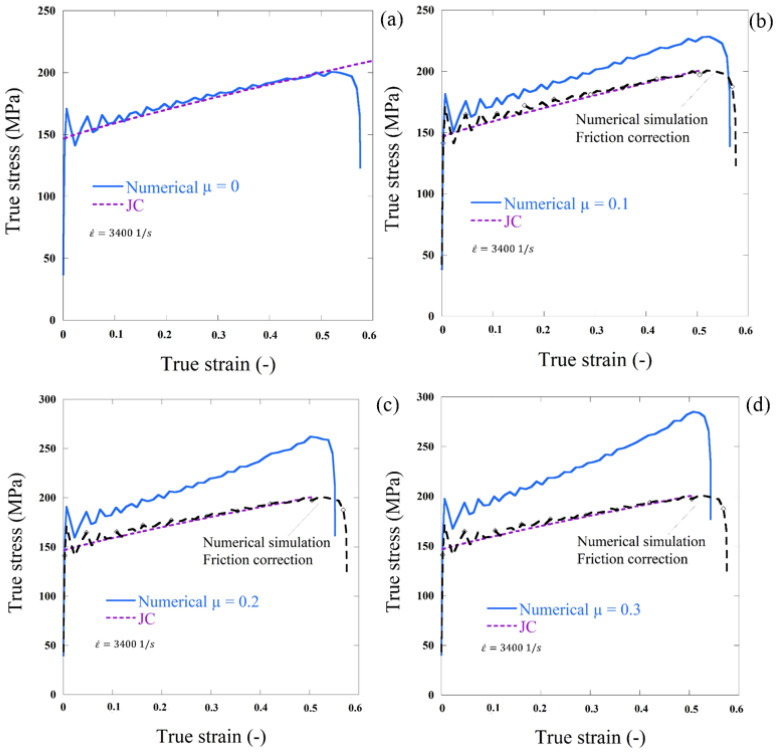
Results of SHPB simulation and (**a**–**d**) comparison between numerical results with Johnson cook model (JC) model for different friction coefficients using the cylinder sheet. $\dot{\varepsilon }=3400\;1/s$.

The Figure 11 reveals that the coefficient of friction greatly influenced the strain pulses that were expressed and transmitted. The maximum reflected strain value was reported in the lubricant contact state specimen (μ = 0) and decreased by approximately 11% as the coefficient of friction rose to μ = 0.2, as seen in Figure 9. Unlike the amount of strain transmitted, which grew with friction. On the other hand, the transmitted strain frequency with μ = 0.3 was around 10% higher than that of the lubricant state.

The elastic waves were evaluated at the same locations during the numerical simulation as in the experiment, and the results of these simulations were presented based on signals from various friction coefficients (incident, transmitted and reflected). The friction coefficient is defined at the beginning and considered to be null (μ = 0).

Using μ = 0, the stress was associated with that expected in the JC model and allocated to the specimen being examined (Figure 11d). Using MATLAB tools, the elastic waves were evaluated by an algorithm that enabled accurate measurement. For varying coefficients of friction μ (0.1, 0.2 and 0.3; Figure 11a–d), the next step was to carry out the same simulation. The coefficient of friction, as stated in [34], increases the degree of stress compared to the case of μ = 0, Equation (12). The results of the dynamic cases of friction correction comparison are shown in Figure 11a–d.

The stresses correction in the last case was approximately 55 MPa. The blue lines correspond to uncorrected behavior determined by an algorithm using MATLAB software directly from the simulations. The red lines reflect the input behavioral material assigned to the material being evaluated (JC model). After friction effect correction, the black lines were from numerical simulations.

**Figure 12:**
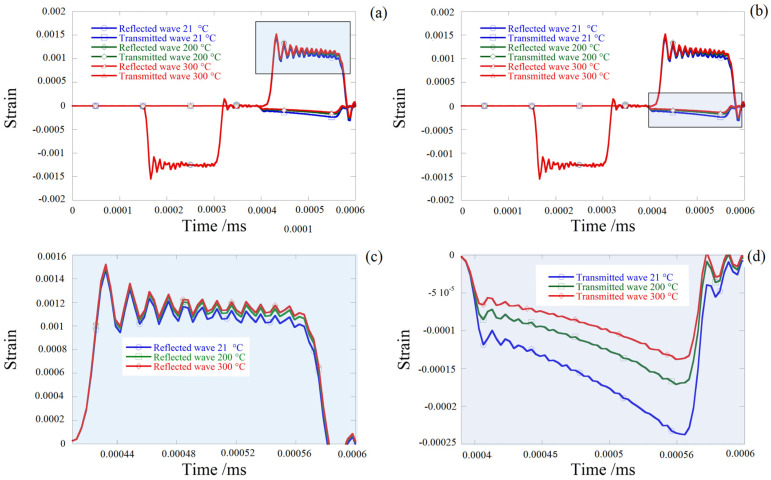
Comparison of reflected and transmitted waves under different temperatures (cylinder sheet). (**a**,**b**) reflected and transmitted waves (**c**) reflected strain waves and (**d**) transmitted strain waves.

A numerical simulation of dynamic compression using the SHPB system with a wide range of temperatures to validate this parameter and to predict the behavior of the tested aluminum alloy in different temperatures, 21, 200 and 300 °C, was performed, and the results are shown in Figure 12.

## 6. Experimental Results (Validation of Cylinder Sheet Specimen in the SHPB System)

**Figure 13:**
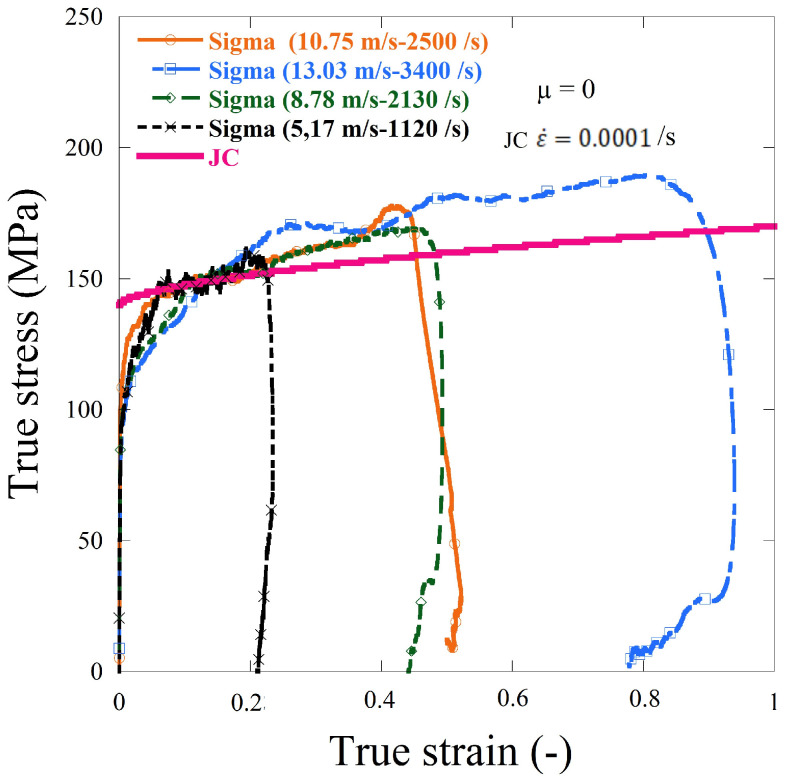
Experimental dynamic compression results using the split Hopkinson pressure bar (HSPB) system, curves without friction correction.

Cylinder sheet specimens made of aluminum alloy AW 5005 were machined to validate this numerical analysis and were used in the current experimental work to investigate the complex behavior using the SHPB system (Figure 2). The corresponding true stress–strain curves for the aluminum material are shown in Figure 13. It is possible to observe similar compressive behavior. The initial yield stress values and patterns in strain hardening are also shown in the Figure 13.

Figure 13 shows that the separation of the cylinder sheets, which occurred at the later stage of the compressive stress wave, did not impact the dynamic response of the material, and the material was not strain rate sensitive. In this experiment, dynamic compression tests were carried out using split Hopkinson pressure bar (HSPB) and a thermal chamber at 170 °C, the same specimen from the previous test (at room temperature) was used (Figure 4 and Figure 5).

**Figure 14:**
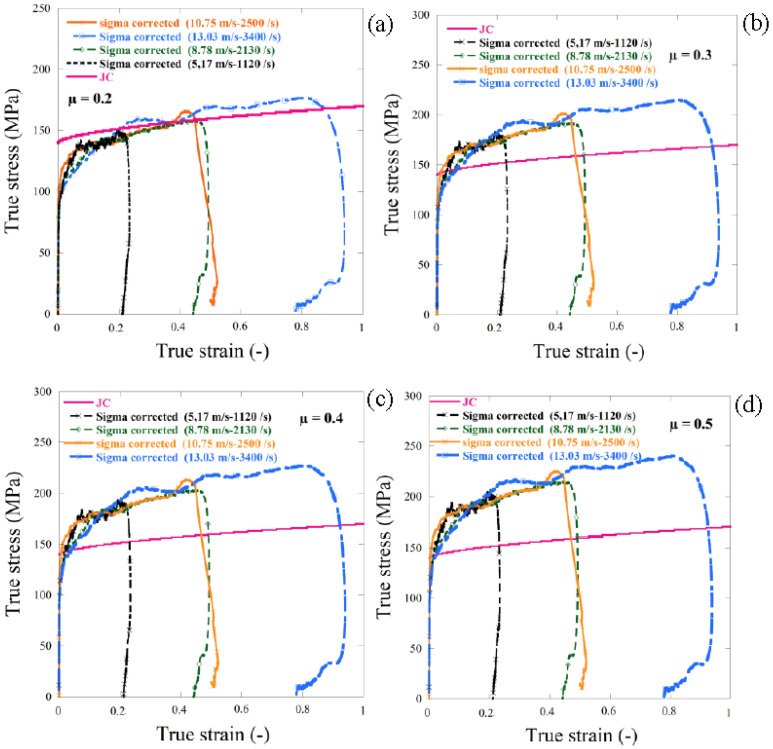
Experimental dynamic compression result (using the HSPB system) curves with different friction corrections. (**a**) µ = 0.2, (**b**) µ = 0.3, (**c**) µ = 0.4 and (**d**) µ = 0.5.

In Figure 14 different friction corrections, from μ = 0.2 to 0.5, was used to analyze the friction effect on the dynamic compression experimental results, it can be observed that the friction coefficient μ = 0.2 gave a good correction as shown in Figure 14a, there was good agreement between the JC model and the dynamic experimental results obtained using the SHPB system at room temperature. The same friction correction was used in the next section to correct the experimental results carried out using HSPB using the thermal chamber at 170 °C.

**Figure 15:**
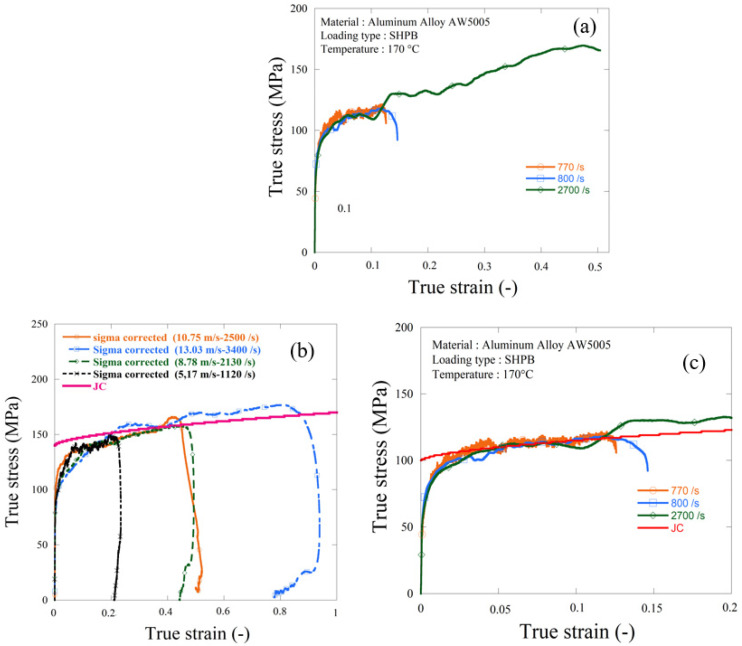
Experimental dynamic compression results using the HSPB system, (**a**) HSPB curves using thermal conditions 170 °C, (**b**) HSPB at room temperature and (**c**) HSPB curves using thermal conditions with the JC model.

It can be observed from Figure 15 that the material was temperature sensitive as the elastic limit was temperature dependent, since in ambient temperature the elastic limit was equal to 147 MPa. However, when the same test was repeated using the thermal chamber at 170 °C it was found that the elastic limit decreased to 115 MPa, which gave a decrease of 18%. It also shows that the material was not always strain rate sensitive even with different thermal conditions.

## 7. Conclusions

In this work, some significant notes were shown experimentally under impact loading. It was found that the model proposed by Klepaczko and Malinowski [34,35,42] could be employed under quasi-static and dynamic loading compression to simply correct the friction effect. Nevertheless, according to the geometry of the specimen used for the experiments, it is important to have accurate knowledge of its boundaries.

For SHPB tests, the preferable dimensions of specimens that guarantee the proper physical law estimation were suggested. Taking a ratio specimen of $S_{0}$ = L_0_/∅ _0_ = 0.5, coupled to a reduced height, L_0_ allowed us to reach large deformation under dynamic loading and high strain rates.

In this study, finite element simulations and experimental studies were performed to investigate the effect of the interface friction, temperature and specimen configuration on the dynamic response of SHPB compression test materials. Simulation of cylindrical specimen geometries with one and four layers and a range of friction coefficients of the specimen/bar interface between 0.0 and 0.3 with temperatures between 21 and 300 °C were considered, the next conclusions can be drawn:

- The friction at the interface between the incident/transmission and specimen bars greatly affected the accuracy of the SHPB test results.
- The friction of the interface changed the stress state in the SHPB specimen. Additionally, the mechanical property of the aluminum alloy could be varied from its true value.
- Similar dynamic responses were seen in experimental results for cylinder specimens and cylinder sheets. Cylinder bonded layers could therefore be regarded as a stable alternative composition for enhancing the control of thin sheet material experiments.

## Figures and Tables

**Table 1:** Definition of the devices used to deliver the range of strain rates in mechanical testing.

$\mathrm{Strain}\;\mathrm{Rate}\;s^{-1}$	$10^{-8}$	$10^{-6}$	$10^{-4}$	$10^{-2}$	$10^{0}$	$10^{2}$	$10^{2}$	$10^{4}$	$10^{4}$	$10^{6}$
**State**	**Isothermal**				**Quasi** **-isothermal/adiabatic**					
	**Without inertia effect**				**With inertia effect**					
**Set-Up**	**Creep**		**Construction, excavation**		**Earthquake, vehicular crash test**		**Impact, explosion**		**Nuclear explosion**	
	**Specialized Hydraulic machines**		**Servo-hydraulic machines**		**Pneumatic hydraulic machines**		**Split Hopkinson pressure bar**		**Impact loading**	
	**Creep**		**Quasi-Static**		**Intermediate strain rate**		**High strain rate**		**Very high strain rate**	

**Table 2:** Chemical properties of the EN AW 5005 aluminum alloy [20].

**Chemical Composition %**	**Fe**	**Si**	**Cu**	**Mn**	**Mg**	**Zn**	**Cr**	**Al**
	0.45	0.3	0.05	0.15	0.5–1.1	0.2	0.1	Balance

**Table 3:** Mechanical properties of the EN AW 5005 aluminum alloy [20].

**Mechanical Properties**	**Yield Strength** **(MPa)**	**Tensile Strength** **(MPa)**	**Elongation** **(%)**	**Hardness** **(HV)**
	45	110	15	32

**Table 4:** Material parameters for the Johnson Cook (JC) model [21].

A (MPa)	B (MPa)	n (-)	C (-)	m (-)	$\dot{\varepsilon }$ $\;(s^{-1})$	$T_{m}\;(K)$	$T_{0}\;(K)$
147	60	0.9	0.003	1.08	1	933	300

**Table 5:** Parameters for failure models [21].

Failure Model Aluminum Alloy (AW 5005)			
H (-)	I (-)	J (-)	K (-)
−0.398	1.452	1.0085	0.6647
